# Gender-differences in imaging phenotypes of osteoarthritis in the osteoarthritis initiative

**DOI:** 10.1038/s41598-025-90782-x

**Published:** 2025-02-20

**Authors:** Virginie Kreutzinger, Katharina Ziegeler, Gabby B. Joseph, John A. Lynch, Nancy E. Lane, Charles E. McCulloch, Michael Nevitt, Thomas M. Link

**Affiliations:** 1 Department of Radiology and Biomedical Imaging, University of California, San Francisco, CA USA; 2 Department of Medicine, Center for Musculoskeletal Health, University of California, Davis, Sacramento, CA USA; 3 Department of Epidemiology and Biostatistics, University of California, San Francisco, CA USA

**Keywords:** Knee osteoarthritis, Magnetic resonance imaging, Sex-differences, Magnetic resonance imaging, Diagnosis

## Abstract

In osteoarthritis (OA) research it is increasingly recognized that stratification according to disease phenotypes is essential for optimizing treatment regimens. Gender-specific differences in clinical OA manifestations have been identified, and this analysis aimed to assess whether these differences extend to imaging phenotypes.

From the Osteoarthritis Initiative database 2523 participants (1409 women and 1114 men) with completed 3T MRI and whole-organ magnetic resonance imaging scores (WORMS) of the right knee at baseline were included. Imaging phenotypes were assigned based on modified Rapid OsteoArthritis MRI Eligibility Score: the inflammatory, meniscus-cartilage, and bone phenotype. Logistic regression was performed to investigate the effect of gender on phenotype, independent of BMI, race, Kellgren & Lawrence (KL) grade and level of physical activity. Female gender was independently associated with lower odds for the meniscus-cartilage (OR 0.61, 95%CI 0.47–0.80, *p* < 0.001) but not for the inflammatory (OR 1.04, 95%CI 0.89–1.24, *p* = 0.697) or the subchondral bone phenotype (OR 1.13, 95%CI 0.95–1.36, *p* = 0.166). This difference highlights an opportunity for future refinements to better accommodate gender/sex differences in disease trajectories while investigating different treatment regimes in knee OA.

**Supplementary Information:**

The online version contains supplementary material available at 10.1038/s41598-025-90782-x.

## Introduction

Osteoarthritis (OA) is a chronic condition, incurring substantial economic burden^1^, both directly through treatment and indirectly through lost work productivity in affected individuals^2^. The societal impact is profound, as OA accounts for more disability cases in the elderly than any other disease^3^. Nevertheless, despite the considerable efforts undertaken to identify effective disease-modifying OA drugs (DMOADs), therapeutic advances in non-surgical treatments remain limited^4,5^. It has been difficult to identify therapeutic targets in OA as the disease results from a complex interplay of different tissues (cartilage, synovium, subchondral bone)^6^. Thus, the research community now focuses on the identification of different disease endo- and phenotypes with imaging as a potentially effective tool for the objective classification of patients into phenotype groups^7^. This approach aims to minimize unsatisfactory DMOAD trial outcomes, by targeting patients with the highest likelihood to benefit from the investigated agent, i.e. anti-inflammatory agents for patients with predominant synovitis. Roemer et al. introduced an MRI-based score, the Rapid OsteoArthritis MRI Eligibility Score (ROAMES)^8^, that defined five distinct imaging phenotypes: the inflammatory phenotype, the meniscus-cartilage phenotype, the subchondral bone phenotype, and the hypertrophic and atrophic phenotypes. Of these image-based phenotypes, the inflammatory, meniscus-cartilage and subchondral phenotypes are considered as the most promising for targeted trials^9^, both because of their relatively higher prevalence^10^, and because their respective proposed underlying pathomechanisms offer promising therapeutic targets^11^.

A different dimension of this trend towards personalized treatment in OA is the increasing awareness of and research on sex-differences^12,13^. Women experience higher OA incidence rates than men, particularly after the age of 50 ^14^. This discrepancy is largely attributed to biological factors, including hormonal differences that may influence cartilage metabolism and joint health^15^. Estrogen is thought to play a protective role in joint health, with its decline during menopause correlating with an increase in OA prevalence among women^16^. OA of the knee is more common in women compared to men across various definitions of OA, whereas no gender disparities are observed in hip and hand OA^17^. Moreover, biomechanical factors such as bone shape^18^, and muscle strength and composition^19^ and injury due to ligament laxity^20^ are thought to contribute to the gender disparities observed in knee OA^12^. Women generally exhibit different knee joint biomechanics compared to men, including wider pelvises relative to knee alignment and differences in ligament laxity, which may predispose them to higher rates of joint wear and tear^21^. These biomechanical differences not only affect the load distribution across the knee joint but also potentially accelerate the progression of OA in women compared to men^22^.

Limited research has thus far been undertaken to understand how these gender-differences may translate into differences in imaging presentations of OA in general, and specifically to imaging phenotypes. As phenotyping based on imaging may be increasingly used in the stratification of patients in DMOAD trials, investigating possible gender-differences becomes important to potentially optimize patient selection for clinical trials.

The objective of this study was to investigate if assignment to currently proposed phenotypes in knee OA differs between men and women and to explore how any disparities might be explained by gender-specific variations in the extent and distribution of MRI-detected lesions, with a special focus on individuals with mild to moderate OA. This understanding could potentially inform more tailored phenotype definitions in the future as well as treatment and management strategies. Our hypothesis at the outset of the study was that any existence of differences in phenotype prevalence between the sexes was explained by demographic factors.

## Results

### Demographics

**Fig. 1:**
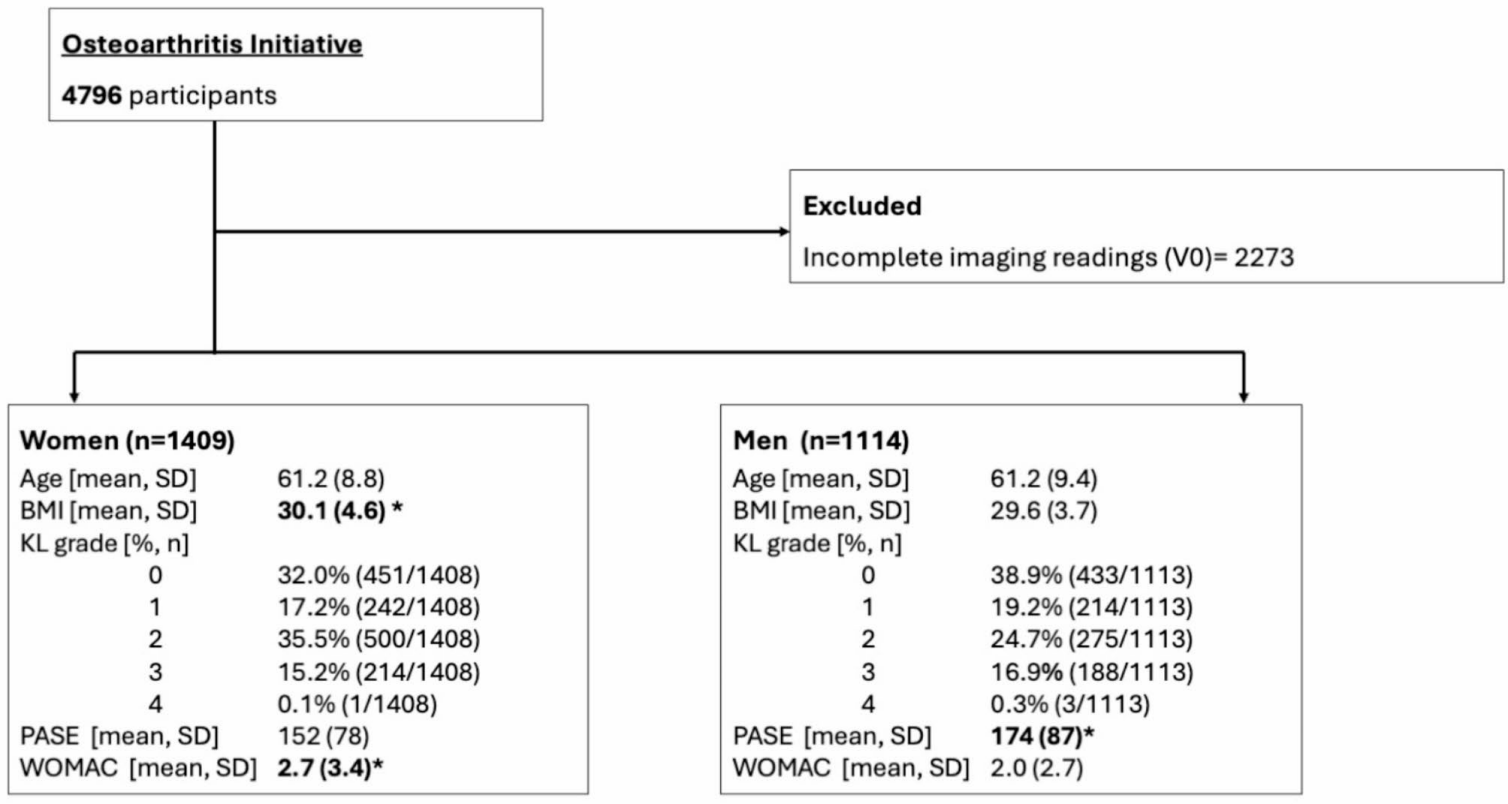
Participant selection and clinical characteristics. V0 = baseline. BMI = Body mass index (kg/m2). KL = Kellgren & Lawrence. PASE = Physical activity score for the elderly. WOMAC = Western Ontario and McMaster Universities OsteoArthritis Index. Significantly (*p* < 0.05) larger values compared to other gender/sex are in bold and marked with an asterisk (*); p-values were derived from unpaired t-tests and Chi-squared tests.

**Fig. 2:**
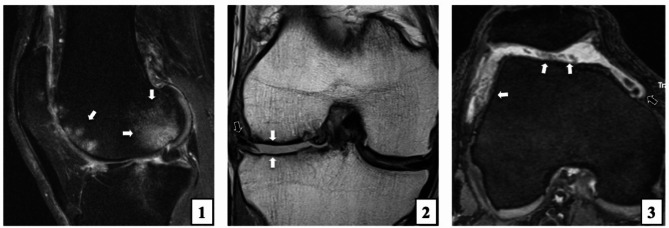
Imaging examples for phenotypes. 1 = bone phenotype: Extensive bone marrow edema-like lesions (white arrows) both in the trochlea and in the lateral femoral condyle. 2 = meniscus-cartilage phenotype: Displaced meniscal tear (black arrow) with widespread full-thickness defects (white arrows) of both femoral and tibial cartilage. 3 = inflammatory phenotype: Effusion synovitis with moderate distension of joint capsule (black arrow) as well as synovial proliferation (white arrows).

After the exclusion of participants without completed imaging or WORMS or synovitis readings, a total of 1114 men and 1409 women were included in this study (see also Fig. 1). Men and women did not differ in mean age (men: 61.2 ± 9.4 years, women: 61.2 ± 8.8 years, *p* = 0.931) but minimally in terms of BMI (men: 29.6 ± 3.7 kg/m^2^, women: 30.1 ± 4.6 kg/m^2^, *p* = 0.004). Distribution of KL grades differed significantly (*p* < 0.001) with men showing a slightly higher prevalence of KL grades 3 & 4 (men: 17.2%, women: 15.3%). Levels of physical activity as measured by the PASE questionnaire were higher in men than women (174 vs. 152, *p* < 0.001), whereas pain (mean WOMAC) was worse in women than in men (2.0 vs. 2.7, *p* < 0.001) (Fig. 2).

### Phenotypes

**Fig. 3:**
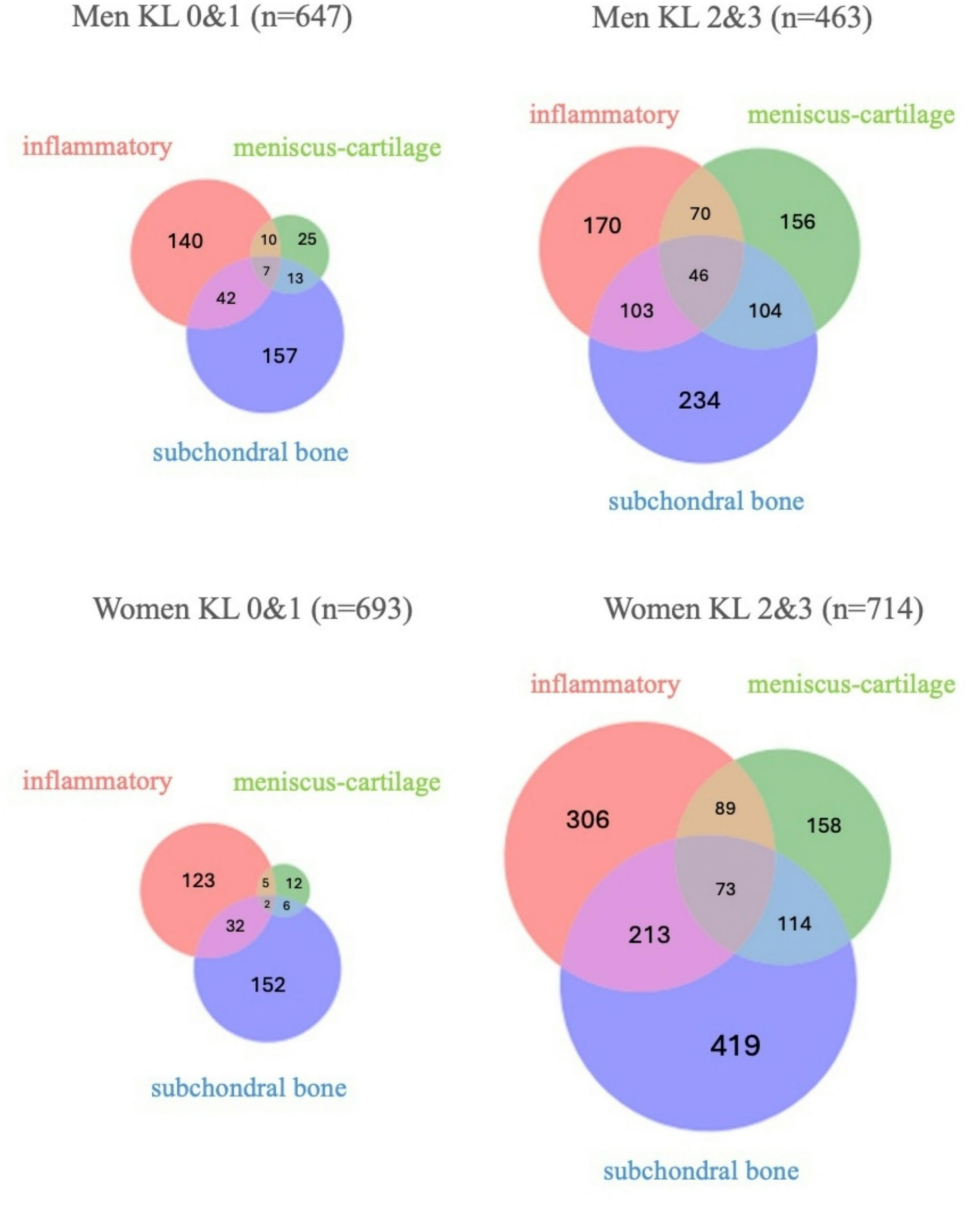
Overlap between phenotypes. Venn diagrams of phenotype overlap with absolute numbers of participants fulfilling respective phenotype criteria, separately for men and women in pre-radiographic OA (KL 0&1) and mild to moderate radiographic OA (KL2&3).

Results of the descriptive analysis of ROAMES criteria by gender are given in Table 1; a subgroup analysis of participants with KL grades 2 & 3 was also performed. The meniscus-cartilage phenotype was fulfilled by more men than women (16.5% vs. 12.1%, *p* = 0.002). Conversely, significantly, more women met the criteria for the bone phenotype (35.5% vs. 40.6%, *p* = 0.008). When only analyzing participants with KL grades 2 & 3 (mild and moderate radiographic OA) percentages increased and the gender-gap widened for the meniscus-cartilage phenotype (33.7% of men vs. 22.1% of women, *p* < 0.001) as well as for the bone phenotype (50.5% of men vs. 58.7% of women, *p* = 0.006) and for the inflammatory phenotype, which was significantly more frequent in women (36.7% vs. 42.9%, *p* = 0.035). Logistic regression showed that women had lower odds of meeting criteria for the meniscus-cartilage phenotype (OR 0.61, 95%CI 0.47–0.80, *p* < 0.001) but not for the inflammatory phenotype (OR 1.04, 95%CI 0.89–1.24, *p* = 0.697) and the subchondral bone phenotype (OR 1.13, 95%CI 0.95–1.36, *p* = 0.166) independent of age, BMI, race, physical activity, and KL grade. In our study population, which also includes individuals at risk for OA and without radiographic OA (KL 0 &1), 54.9% (1384/2523) participants met the criteria for any of the investigated phenotypes while in the subset of participants with KL grades of 2 & 3 this number increased to 73.8% (869/1177). No gender differences in these proportions were found. Many of the participants met the criteria for more than one phenotype, indicating a significant overlap. For example, 33.4% (140 out of 419) of the women who met the criteria for the subchondral bone phenotype in the KL 2&3 group also met the criteria for both subchondral and inflammatory phenotypes. Furthermore, 10.2% (73 out of 713) of women with KL 2&3 met the criteria for all three phenotypes. A graphical representation of the overlap between phenotypes is given in Fig. 3.

**Table 1:** Frequencies of phenotypes.

		Men	Women	*p*
All participants	Inflammatory phenotype	28.0 (312/1114)	30.4 (429/1409)	0.181
	Meniscus-cartilage phenotype	**16.5 (184/1114)**	12.1 (171/1409)	0.002
	Subchondral bone phenotype	35.5 (395/1114)	**40.6 (572/1409)**	0.008
KL 2 and 3	Inflammatory phenotype	36.7 (170/463)	**42.9 (306/714)**	0.035
	Meniscus-cartilage phenotype	**33.7 (156/463)**	22.1 (158/714)	< 0.001
	Subchondral bone phenotype	50.5 (234/463)	**58.7 (419/714)**	0.006

KL = Kellgren & Lawrence. P values derived from Chi-squared tests.

### Meniscal tears

In addition to the phenotypes, tissues were also analyzed separately. The distribution of meniscal tears of different severities is shown in Table 2. In the medial meniscus the posterior horn was most affected by tears in both men and women, but men had significantly higher rates (43.4% vs. 22.0%, *p* < 0.001). Men also displayed more meniscal tears in the body and posterior horn of the medial meniscus. In the lateral meniscus the trend was less clear. While in the anterior horn men and women had similar overall rates of tears (11.2% vs. 10.9%) the distribution regarding severity differed significantly (*p* = 0.003) with women showing more displaced tears (3.8% vs. 2.0%) and men showing more macerations (2.0% vs. 1.1%). Similar to the findings in the medial meniscus, men exhibited significantly (*p* = 0.003) more tears in the posterior horn of the lateral meniscus with 16.5% vs. 12.3% in women. Additionally, frequencies of meniscal tears in the subgroup of participants with KL grades 2 & 3 were investigated and are given as a supplementary table. This subgroup showed a very similar distribution, with a male predilection for any kind of tear in the medial meniscus, and in the lateral meniscus only for high grade tears, with a female predilection for less complex tears. As expected, participants with KL grades 2 & 3 exhibited overall higher frequencies of meniscal tears than the cohort as a whole.

**Table 2:** Frequencies of WORMS meniscus gradings.

			none	Intrasubstance degeneration	Simple Tear	Displaced Tear	Maceration	*p*
Lateral Meniscus	Anterior Horn	M	81.7 (910/1114)	6.9 (77/1114)	7.7 (86/1114)	2.0 (22/1114)	2.0 (22/1114)	**0.003**
		F	79.3 (1118/1409)	8.7 (122/1409)	7.0 (99/1409)	3.8 (54/1409)	1.1 (16/1409)	
	Body	M	76.0 (847/1114)	6.6 (73/1114)	11.4 (127/1114)	3.9 (43/1114)	2.2 (24/1114)	0.395
		F	74.8 (1054/1409)	7.7 (109/1409)	11.4 (161/1409)	4.6 (65/1409)	1.4 (20/1409)	
	Posterior Horn	M	73.2 (815/1114)	10.3 (115/1114)	10.6 (118/1114)	4.3 (48/1114)	1.6 (18/1114)	**0.016**
		F	76.1 (1072/1409)	11.6 (163/1409)	8.8 (124/1409)	2.8 (40/1409)	0.7 (10/1409)	
Medial Meniscus	Anterior Horn	M	93.4 (1040/1114)	2.2 (24/1114)	1.3 (15/1114)	1.7 (19/1114)	1.4 (16/1114)	**< 0.001**
		F	96.5 (1360/1409)	2.1 (30/1409)	0.7 (10/1409)	0.4 (5/1409)	0.3 (4/1409)	
	Body	M	55.1 (614/1409)	8.9 (99/1114)	14.7 (164/1114)	14.7 (164/1114)	6.6 (73/1114)	**< 0.001**
		F	70.6 (995/1409)	11.6 (163/1409	8.8 (124/1409)	7.1 (100/1409)	1.9 (27/1409)	
	Posterior Horn	M	39.0 (434/1114)	17.7 (197/1114)	22.9 (255/1114)	15.1 (168/1114)	5.4 (60/1114)	**< 0.001**
		F	51.5 (725/1409)	26.5 (373/1409)	14.5 (205/1409)	6.1 (86/1409)	1.4 (20/1409)	

M = men. F = women. Relative and absolute frequencies of different types of meniscal tears per investigated location in men vs. women. P values were derived from chi-squared tests.

### Cartilage lesions

Cartilage damage in the patellofemoral joint (PFJ) was significantly more prevalent in women than in men. The patellar cartilage showed no abnormalities in 31.2% of men but only in 16.5% of women. This significant (*p* < 0.001) difference in distribution continued towards higher grade cartilage lesions (WORMS grade 5 and 6) with 30.5% of women vs. only 15.8% of men showing defects of this magnitude. Similar but less pronounced differences were seen at the trochlea with high-grade cartilage lesions in 13.1% of men vs. 13.7% of women (*p* < 0.001). In contrast to this distribution, higher-grade cartilage lesions at the medial tibia were more common in men (4.3% vs. 2.6%, *p* = 0.016). The distribution of cartilage lesions is shown in Table 3. When summarizing cartilage lesions into a sum score per participants, women exhibited significantly higher overall cartilage damage (6.9 vs. 8.1, *p* < 0.001).

**Table 3:** WORMS cartilage grade distribution.

		0	1	2	2.5	3	4	5	6	*p*
Patella	M	31.2 (348/1114)	17.3 (193/1114)	15.2 (169/1114)	3.3 (37/1114)	15.1 (168/1114)	2.1 (23/1114)	13.8 (154/1114)	2.0 (22/1114)	**< 0.001**
	F	16.5 (232/1409)	12.0 (169/1409)	12.0 (169/1409)	3.8 (54/1409)	19.5 (275/1409)	5.7 (80/1409)	23.6 (333/1409)	6.9 (97/1409)	
Trochlea	M	44.8 (499/1114)	14.2 (158/1114)	10.9 (121/1114)	3.0 (33/1114)	13.3 (148/1114)	0.8 (9/1114)	12.2 (136/1114)	0.9 (10/1114)	**< 0.001**
	F	38.7 (545/1409)	15.0 (211/1409)	13.6 (191/1409)	3.0 (42/1409)	11.6 (164/1409)	1.5 (21/1409)	13.7 (193/1409)	3.0 (42/1409)	
Lateral femur	M	74.8 (833/1114)	5.7 (63/1114)	8.7 (97/1114)	1.3 (15/1114)	5.7 (64/1114)	0.1 (1/1114)	3.4 (38/1114)	0.3 (3/1114)	0.127
	F	70.5 (993/1409)	8.2 (116/1409)	9.7 (137/1409)	1.6 (22/1409)	6.1 (86/1409)	0.4 (6/1409)	3.1 (44/1409)	0.4 (5/1409)	
Lateral tibia	M	56.0 (624/1114)	17.6 (196/1114)	13.6 (152/1114)	2.6 (29/1114))	5.9 (66/1114)	0.3 (3/1114)	3.3 (37/1114)	0.6 (7/1114)	0.400
	F	51.2 (722/1409)	20.3 (286/1409)	14.7 (207/1409)	2.4 (34/1409)	7.0 (98/1409)	0.4 (6/1409)	3.2 (45/1409)	0.8 (11/1409)	
Medial Femur	M	52.7 (587/1114)	5.5 (61/1114)	16.9 (188/1114)	2.2 (24/1114)	14.4 (160/1114)	0.5 (6/1114)	7.7 (86/1114)	0.2 (2/1114)	0.172
	F	51.7 (729/1409)	7.6 (107/1409)	16.7 (236/1409)	2.1 (29/1409)	14.5 (205/1409)	1.2 (17/1409)	6.0 (84/1409)	0.1 (2/1409)	
Medial tibia	M	78.4 (873/1114)	3.6 (40/1114)	4.8 (54/1114)	1.6 (18/1114)	6.6 (73/1114)	0.7 (8/1114)	4.0 (45/1114)	0.3 (3/1114)	**0.016**
	F	80.9 (1140/1409)	4.2 (59/1409)	4.0 (56/1409)	0.8 (11/1409)	5.7 (81/1409)	1.8 (25/1409)	2.4 (34/1409)	0.2 (3/1409)	

M = men. F = women. Relative and absolute frequencies of different types of cartilage lesions per investigated location in men vs. women. P values were derived from chi-squared tests. 0 = normal thickness and signal. 1 = T2 signal abnormality. 2 = partial thickness lesion < 1 cm. 2.5 = full thickness lesion < 1 cm. 3 = multiple < 1 cm partial defects or a single lesion > 1 cm but < 75%. 4 = diffuse partial thickness loss ≥ 75% of region. 5 = multiple areas of full thickness loss > 1 cm but < 75% of region or full thickness < 1 cm and partial thickness or multiple full thickness < 1 cm. 6 = diffuse full thickness loss ≥ 75% of region.

### Bone marrow lesions

Following the trend observed in cartilage lesions, women showed more bone marrow lesions in the PFJ while men showed more in the medial femorotibial joint (MFT). Like the cartilage lesions this was most pronounced in the patella where 36.6% of men vs. 51.4% of women (*p* < 0.001) showed any type of lesion. In the medial tibia 15.1% of men exhibited bone marrow lesions but only 10.7% of women (*p* < 0.001). The distribution of bone marrow lesions is detailed in Table 4.

**Table 4:** WORMS-Bone marrow lesion distribution.

		None	< 5 mm	5–20 mm	> 20 mm	*p*
Patella	M	63.4 (706/1114)	10.6 (118/1114)	21.0 (234/1114)	5.0 (56/1114)	**< 0.001**
	F	48.7 (686/1409)	13.5 (190/1409)	29.5 (415/29.5)	8.4 (118.1409)	
Trochlea	M	69.3 (772/1114)	6.8 (76/1114)	17.3 (193/1114)	6.6 (73/1114)	**0.013**
	F	64.3 (906/1409)	9.8 (138/1409)	17.9 (252/1409)	8.0 (113/1409)	
Lateral femur	M	93.2 (1038/1114)	2.0 (22/1114)	3.4 (38/1114)	1.4 (16/1114)	0.712
	F	92.3 (1300/1409)	2.3 (33/1409)	4.1 (58/1409)	1.3 (18/1409)	
Lateral tibia	M	87.8 (978/1114)	4.9 (55/1114)	5.9 (66/1114)	1.3 (15/1114)	0.894
	F	88.5 (1247/1409)	4.7 (66/1409)	5.3 (75/1409)	1.5 (21/1409)	
Medial femur	M	83.2 (927/1114)	6.6 (73/1114)	8.0 (89/1114)	1.1 (25/1114)	0.078
	F	85.7 (1207/1409)	5.7 (80/1409)	7.6 (107/1409)	1.1 (15/1409)	
Medial tibia	M	84.9 (946/1114)	3.0 (33/1114)	8.7 (97/1114)	3.4 (38/1114)	**< 0.001**
	F	89.3 (1258/1409)	3.3 (46/1409)	6.2 (88/1409)	1.2 (17/1409)	

M = men. F = women. Relative and absolute frequencies of bone marrow lesions per investigated location in men vs. women. P values were derived from chi-squared tests.

### Synovitis

Overall synovitis, expressed as a mean sum score (out of 14), did not differ between men (4.5, SD 2.6) and women (4.3 SD 2.5) (*p* = 0.082), but results in different sub-categories of MRI-detected inflammation were heterogeneous. Women had significantly higher grades of effusion synovitis when using the ACLOAS method (51.3% vs. 57.3% pathological scores, *p* < 0.001), whereas men showed significantly higher scores for both categories of Hoffa’s fat pad changes. No differences were found in effusion-synovitis measured by MOAKS and synovial proliferation score. Frequencies of different scores in men and women are detailed in Table 5.

**Table 5:** Synovitis grades.

		0	1	2	3	*p*
ACLOAS	M	48.7 (543/1114)	36.5 (407/1114)	12.7 (141/1114)	2.1 (23/1114)	**< 0.001**
	F	42.4 (598/1409)	42.2 (595/1409)	14.3 (202/1409)	1.0 (14/1409)	
MOAKS	M	17.7 (197/1114)	61.8 (689/1114)	17.1 (191/1114)	3.3 (37/1114)	0.079
	F	20.8 (293/1409)	57.2 (806/1409)	19.0 (268/1409)	3.0 (42/1409)	
Hoffa’s intensity	M	18.9 (210/1114)	37.6 (419/1114)	36.4 (406/1114)	7.1 (79/1114)	**0.007**
	F	23.4 (330/1409)	34.8 (491/1409)	32.9 (463/1409)	8.9 (125/1409)	
Hoffa’s fat pad size	M	18.5 (206/1114)	57.0 (635/1114)	20.7 (231/1114)	3.8 (42/1114)	**< 0.001**
	F	23.4 (330/1409)	64.3 (906/1409)	11.4 (161/1409)	0.9 (12/1409)	
Synovial proliferation score	M	71.6 (798/1114)	23.2 (259/1114)	5.1 (57/1114))		0.260
	F	68.8 (969/1409)	26.0 (367/1409)	5.2 (73/1409)		

M = men. F = women. ACLOAS = Anterior Cruciate Ligament OsteoArthritis Score. MOAKS = MRI Osteoarthritis Knee Score. Relative and absolute frequencies of synovitis grades in men vs. women. P values were derived from chi-squared tests.

## Discussion

Phenotyping based on imaging is a promising approach to better capture the complex and multi-facetted nature of knee OA with the goal to better stratify study subjects for enrollment in clinical DMOAD trials. While different imaging phenotypes have been proposed, the most widely accepted are the meniscus-cartilage, the inflammatory, and the subchondral bone phenotype. To the best of our knowledge, this study is novel in that we evaluated the prevalences of the different phenotypes by gender/sex. Men had a higher prevalence of the meniscus-cartilage phenotype and women of the inflammatory and the subchondral bone phenotypes.

The meniscus-cartilage phenotype is characterized by severe damage to the meniscus, which is accompanied by a widespread loss of cartilage. Multiple studies have established that lesions in the meniscus can lead to decreased shock absorption and asymmetrical load distribution, accelerating incident cartilage loss in a predominantly mechanical manner^23,24^. In our analysis, women had almost 50% lower odds for the meniscus cartilage phenotype independent of KL, BMI, age and PASE. A possible explanation for these disparities is the marked difference in the prevalence of meniscal tears, especially higher-grade tears of the medial meniscus, which were substantially more prevalent in men than in women. Our findings regarding meniscal tears are supported by previous studies that found slightly higher numbers of meniscal tears in men after ACL reconstruction^9,25,26^, but somewhat diverge from findings by Hong et al., who found higher frequencies of meniscal tears in women in a cohort of individuals with occupationally increased mechanical loading of the knee joints^27^. In addition to the described trends in meniscal tears, differences in the location of cartilage lesions may also have contributed to the observed differences in fulfillment of the criteria for this phenotype: men exhibited slightly more cartilage damage in the medial FT joint, while the PFJ (which does not factor into this phenotype) was predominantly affected in women. It is established that men are more likely than women to exhibit varus malalignment^28^ – thus, a slightly higher mechanical load is transmitted to the medial FT joint in men, and consecutively, more lesions are expected. Also, men exhibited marginally higher levels of physical activity than women in our study population, which may partially explain the higher rates of meniscal tears, although this could not be further investigated, as detailed information on past sports injuries are not available in the OAI dataset.

In contrast to the more mechanically driven meniscus-cartilage phenotype, the inflammatory phenotype is hypothesized to be driven by low-grade synovitis^29^. Synovitis in OA is significantly associated with accelerated progression of structural imaging outcomes and is a promising therapeutic target considering the wide array of anti-inflammatory agents available for other inflammatory arthropathies^9^. In participants with mild to moderate radiographic OA (i.e. patients most likely to be eligible for inclusion in clinical trials) significantly more women than men exhibited the inflammatory phenotype. However, regression analysis showed that the association of female gender and inflammatory phenotype was not independent of age, BMI, race, physical and baseline KL grade. Other studies have reported higher inflammatory activity in women with OA^30^ and there are well-established gender differences in levels of inflammation in a wide array of autoimmune diseases^31,32^. However, synovial inflammation may be less straightforward to detect and quantify on MR imaging (especially without contrast-enhancement) than pathologies in other tissues – not only because of the undulating nature of synovitis in OA, which may be missed outside the period of active inflammation, but also due to the diverse imaging surrogate biomarkers that have been proposed^33^. In our cohort, effusion synovitis was more common in women, synovial proliferation was seen in similar proportions in men and women while Hoffa’s synovitis was more common in men. The latter finding is especially interesting, because cartilage damage in the PFJ was more pronounced in women, but PFJ OA has been linked to abnormalities of Hoffa’s fat pad (especially the supero-lateral part)^34^ - further research is needed to reconcile these observations.

While both the meniscus-cartilage and the inflammatory phenotype are driven by pathological processes within the joint space, the bone phenotype is hypothesized to stem from disturbances in bone homeostasis, both by altered bone metabolism and reaction to biomechanical loading. These changes may cause cartilage loss by impacting cartilage-bone crosstalk at the bone-cartilage interface^35^, and conversely also be the result of impaired force-dispersion due to cartilage damage^36^. In our cohort, the subchondral bone phenotype was significantly more prevalent in women, but, similar to the inflammatory phenotype, this association was not independent of demographic factors, KL grade, and physical activity. This independence of gender is somewhat surprising, considering the strong evidence for a female predisposition to disturbances in bone metabolism with decreasing estrogen levels after menopause^37^, to which the substantial increase in OA incidence in women after menopause may be partially attributed^14^.

Imaging phenotypes are not mutually exclusive, and our data indicates an increasing overlap of criteria fulfillment with increasing KL grades, which may be understood as a convergence of imaging phenotypes with disease progression. For the purposes of stratification into clinical trials, good separation between phenotypes, while simultaneously capturing a sufficient number of patients is essential. While the phenotype criteria proposed in this investigation achieve the second aim by capturing 73.8% of participants with mild to moderate radiographic OA, there is substantial overlap between phenotypes. Furthermore, both synovitis and BMELL are not necessarily progressive and may well recede with time, which would be desirable to address in future refinements of these criteria, alongside the phenotype overlap.

Additional to the discussed impact on DMOAD trial inclusion, some of our exploratory findings on lesion distribution are important with regard to trial endpoints. The European Medicines Agency (EMA) recommends in their currently effective guideline on disease-modifying drugs in OA^38^ that joint space narrowing assessed on weight bearing AP views should be used to assess structural damage. This metric is best suited to assess patients in whom the medial FT joint is most severely affected, a state for which our data indicate a slight male predominance. A continuation of this approach can be found in modern DMOAD trials: Regardless of proposed mechanisms of drug action, quantitative MRI measurements of the medial femoral cartilage are used as endpoints, for example in the evaluation of ADAMTS-5 inhibitors (NCT03595618) or Cathepsin K inhibitors (NCT02705625) in symptomatic knee OA. With this convenient, yet narrow focus on just one joint region, patients with disease in other compartments (e.g. PFJ), who according to our data are more likely to be female, would demonstrate limited effects of the drug. This highlights the need for tissue specific evaluations with less focus on the location of these changes.

In addition to the implications for treatment of OA, an improved understanding of OA phenotypes is also likely to foster novel insights into the etiology of the disease^39^. As the scientific understanding of OA has evolved in recent decades, away from a simple ‘wear and tear’ concept, towards a more nuanced understanding of metabolic and inflammatory factors, the future of phenotype research may offer chances to better understand the etiology and variety of disease trajectories of OA^40^.

There are some limitations to our study. Firstly, the cross-sectional study design limited the analysis of the longitudinal reproducibility of phenotype assignment. The cohort under investigation also contained individuals that are only at risk of OA or with very early disease, and assigning purely image-based phenotypes in these individuals is less likely to be successful than in those with more severe findings. While this cohort of individuals may be suitable to test general rates of phenotype criteria fulfillment, possibilities for refinement of these criteria are sparse in this context, as the question of their clinical meaningfulness can only be addressed in interventional studies. Lastly, imaging alone can only really define endotypes of the disease. More likely, successful phenotyping would require further dimensions of disease, e.g. clinical, genomic, or metabolic data.

In conclusion, gender differences in OA translated into different frequencies of imaging phenotypes in men and women. In this OAI subgroup, men were more likely to be categorized as a meniscus-cartilage phenotype, while women were more likely to demonstrate both an inflammatory and a subchondral bone phenotype, albeit not independently of other clinical and/or demographic factors. As imaging-derived phenotyping had been proposed for patient stratification in clinical DMOAD trials in OA, these gender differences need to be taken into account to improve recruitment and possibly define more accurate (image-based) outcomes. An example to achieve this might be the inclusion of joint malalignment rather than only meniscal tears in a mechanical phenotype. Further research may focus on multi-dimensional or deep phenotyping, drawing information not only from imaging but also from clinical data.

## Materials and methods

### Participants and study design

All participants from the Osteoarthritis Initiative (OAI) with available semi-quantitative assessments of magnetic resonance imaging (MRI) of the right knee at study enrollment were included and retrospectively analyzed in this cross-sectional study. As this investigation is focusing on differences between men and women in this cohort, a short clarification of terminology is warranted^41^. The original questionnaire of the OAI asked participants to identify their gender (male or female) and not their sex. We have therefore decided to use the term gender, even if biological properties are reported, and sex is the more appropriate term. A flow chart documenting subject selection and demographics is shown in Fig. 1. The OAI is a multi-center longitudinal, observational study involving 4,796 participants, focused on evaluating biomarkers in OA. The dataset al.so included the subjects’ Western Ontario and McMaster Universities Osteoarthritis (WOMAC) pain subscale scores^42^, as well as the Physical Activity Scale for the Elderly (PASE)^43^, which evaluates leisure activities and an expert assessment of knee OA severity on radiographs, i.e. the Kellgren & Lawrence (KL) grade of the right knee. The institutional review boards at each center provided approval for the informed consent documents, study protocols, and any amendments.

### Ethics declaration

All research activities adhered to the principles of the Helsinki Declaration. Institutional review boards (IRB) of each center (the Ohio State University’s Biomedical Science Institutional Review Board; the University of Maryland, Baltimore, Institutional Review Board; the University of Pittsburgh Institutional Review Board; the Memorial Hospital of Rhode Island, Institutional review Board, the Committee on Human Research at the University of California, San Francisco; approval number 10–00532) approved informed consent documentation, study protocols and amendments before study commencement.

### Image acquisition

MR images for all participants were acquired using four identical 3.0 T Siemens MAGNETOM Trio scanners and quadrature transmit-receive coils (USA Instruments, Aurora, OH, USA) across four locations: The Ohio State University, Columbus, OH; University of Maryland School of Medicine, Baltimore, MD; University of Pittsburgh, Pittsburgh, PA; and Memorial Hospital of Rhode Island, Pawtucket, RI. Image analysis was performed using the following sequences: sagittal 3D dual-echo in steady state (DESS) sequence (TR/TE = 16.3/4.7 ms, spatial resolution = 0.365 mm × 0.456 mm, slice thickness = 0.7 mm, flip angle 25°, bandwidth 185 Hz/pixel), sagittal 2D intermediate-weighted (IW) fast spin-echo (FSE) sequence with fat saturation (TR/TE = 3200/30 ms, spatial resolution = 0.357 mm × 0.511 mm, slice thickness = 3.0 mm, flip angle 180°, bandwidth 248 Hz/pixel), coronal 3D fast low angle shot (FLASH) sequence with selective water excitation (TR/TE = 20/7.57 ms, spatial resolution = 0.313 mm × 0.313 mm, slice thickness = 1.5 mm), and coronal 2D IW FSE sequence (TR/TE = 3700/29 ms, spatial resolution = 0.365 mm × 0.456 mm, slice thickness = 3.0 mm, flip angle 12°, bandwidth 352 Hz/pixel). Comprehensive details on these sequences are available in the OAI MR protocol documentation online (https://nda.nih.gov/oai/image-acquisitions).

### Image analysis

#### WORMS grading

Semiquantitative image assessments, required as building blocks for phenotypes in this post-hoc analysis, were performed by trained readers, using established scoring. Thus, baseline MR images of the right knee were selected and imaging changes in menisci, cartilage, and subchondral bone (i.e. bone marrow edema like lesions (BMELL)), were evaluated using the established modified^44^ whole organ MRI score (WORMS)^45^. Meniscal morphology was evaluated in six regions (anterior, body, and posterior of both medial and lateral menisci). The meniscal grading ranged from 0 (normal) to 4 (maceration). Cartilage was graded on an eight-point scale in 6 regions (patella, trochlea, medial femoral condyle, medial tibia, lateral femoral condyle, and lateral tibia), where 0 signifies normal cartilage and subsequent levels indicate increasing severity of defects, with scores up to 6 representing diffuse (> 75%) full-thickness loss. BMELLs were defined as areas of increased T_2_ signal intensity and rated on a four-point scale from 0 (none) to 3 (> 20 mm in diameter); they were also assessed separately in the patella, trochlea, medial femoral condyle, medial tibia, lateral femoral condyle, and lateral tibia. The reproducibility of WORMS assessments has been studied previously and is given as Cohen’s kappa > 0.8^45^.

#### Synovitis

Images were semi-quantitatively evaluated for MR biomarkers of synovial inflammation using five scores: the Anterior Cruciate Ligament OsteoArthritis Score (ACLOAS)^46^, the MRI Osteoarthritis Knee Score (MOAKS)^47^, and assessments of infrapatellar fat pad (IFP) abnormalities in size and signal intensity, along with the synovial proliferation score (SPS)^48^. Effusion-synovitis was rated using ACLOAS on sagittal fs IW images by measuring the suprapatellar recess’s maximum AP diameter and using MOAKS on axial fs IW images with a 4-point scale. IFP Hoffa synovitis was graded from 0 (no signal abnormality) to 3 (signal similar to fluid) based on size and signal intensity^49^. The synovial proliferation score evaluated synovial irregularity as 1 and more extensive villonodular proliferation as 2 ^51^.The reliability of these semi-quantitative assessments has been studied in detail in a previous investigation, demonstrating high reliability of Cohens kappa ≥ 0.84 ^51^.

### Phenotypes

To classify participants into phenotype categories, a modified RapidOsteoArthritis MRI Eligibility Score (ROAMES) was used as detailed below to better account for early disease^8^. Based on this scoring system, three different imaging phenotypes were defined as follows:

*Inflammatory* To meet the criteria for an inflammatory phenotype a combination of at least mild suprapatellar joint effusion (MOAKS^47^ effusion synovitis ≥ 1) and an irregularity of the synovial membrane (proliferation score ≥ 1) were required.

*Meniscus/Cartilage* This category required a displaced tear or maceration of the lateral or medial meniscus (WORMS meniscus grade ≥ 3) accompanied by at least a diffuse partial thickness lesion (WORMS cartilage grade ≥ 3) in the same compartment (medial or lateral femorotibial (FT) joint).

*Subchondral bone* For this phenotype, a sum score of ≥ 3 for BMELLs (out of a maximum of 18) was required; this equates for example to one large lesion (> 20 mm; WORMS grade 3) or three smaller lesions in different joint compartments (< 5 mm; WORMS grade 1).

Imaging examples of these phenotypes are given in Fig. 2.

### Statistical analysis

All analyses were carried out using SPSS Version 29. Baseline demographics of men and women were compared using either T-tests for independent samples or Chi-squared tests, depending on data type. Frequencies of WORMS grades per region as well synovitis scores were compared, and unadjusted rates of phenotype fulfillment were compared using Chi-squared tests. A subgroup analysis of participants with KL grades 2 & 3 was also performed, as patients with similar grades are typically enrolled in clinical trials. Sum scores of lesions were calculated to compare the extent of tissue affection across all evaluated regions. Furthermore, logistic regression was used to assess whether female gender was associated with the presence of each phenotype; regressions were adjusted for age, BMI, race, baseline Kellgren & Lawrence (KL) grade and PASE. Results with two-sided p-values less than 0.05 were considered statistically significant for all statistical tests.

## Electronic supplementary material

Below is the link to the electronic supplementary material.

Supplementary Material 1

## Data Availability

All data and materials presented in this study are available at OAI, https://nda.nih.gov/oai.
