# Supplementary material for: Gender-differences in imaging phenotypes of osteoarthritis in the osteoarthritis initiative
Source: Sci Rep. 2025 Feb 20;15:6219. doi: 10.1038/s41598-025-90782-x (PMC11842562; doi:10.1038/s41598-025-90782-x)
Supplement: Supplementary file 1 — Supplementary Material 1 [file 41598_2025_90782_MOESM1_ESM.docx]

Supplementary Table 1. Frequencies of WORMS meniscus gradings in individuals with mild to moderate radiographic OA (KL grades 2 & 3). M=men. F=women. Relative and absolute frequencies of different types of meniscal tears per investigated location in men vs. women. P values were derived from chi-squared tests.

|  | | | none | Intrasubstance degeneration | Simple Tear | Displaced Tear | Maceration | p |
| --- | --- | --- | --- | --- | --- | --- | --- | --- |
| Lateral Meniscus | Anterior Horn | M | 73.9  (342/463) | 9.3  (43/463) | 9.9  (46/463) | 2.6  (12/463) | 4.3  (20/463) | **0.001** |
|  |  | F | 70.6  (504/714) | 12.8  (91/714) | 8.7  (62/714) | 6.2  (44/714) | 1.8  (13/714) |  |
|  | Body | M | 69.6  (322/463) | 7.3  (34/463) | 11.9  (55/463) | 6.5  (30/463) | 4.8  (22/463) | 0.203 |
|  |  | F | 69.6  (497/714) | 9.5  (68/714) | 11.2  (80/714) | 7.1  (51/714) | 2.5  (18/714) |  |
|  | Posterior Horn | M | 64.8  (300/463) | 12.1  (56/463) | 12.1  (56/463) | 7.6  (35/463) | 3.5  (16/463) | **0.008** |
|  |  | F | 70.0  (500/714) | 14.0  (100/714) | 10.4  (74/714) | 4.2  (30/714) | 1.4  (10/714) |  |
| Medial Meniscus | Anterior Horn | M | 87.9  (407/463) | 3.9  (18/463) | 2.2  (10/463) | 3.0  (14/463) | 3.0  (14/463) | **<0.001** |
|  |  | F | 94.3  (673/714) | 3.1  (22/714) | 1.4  (10/714) | 0.7  (5/714) | 0.6  (4/714) |  |
|  | Body | M | 37.2  (172/463) | 10.4  (48/463) | 16.2  (75/463) | 23,1  (107/463) | 13.2  (61/463) | **<0.001** |
|  |  | F | 59.5  (425/714) | 14.2  (101/714) | 10.6  (76/714) | 11.9  (85/714) | 3.8  (27/714) |  |
|  | Posterior Horn | M | 25.5  (118/463) | 15.8  (73/463) | 24.6  (114/463) | 24.0  (111/463) | 10.2  (47/463) | **<0.001** |
|  |  | F | 45.0  (321/714) | 24.4  (174/714) | 17.1  (122/714) | 10.8  (77/714) | 2.8  (20/714) |  |
